# An Audit of Protocol Deviations Submitted to an Institutional Ethics Committee of a Tertiary Care Hospital

**DOI:** 10.1371/journal.pone.0146334

**Published:** 2016-01-06

**Authors:** Sharmila V. Jalgaonkar, Shruti S. Bhide, Raakhi K. Tripathi, Yashashri C. Shetty, Padmaja A. Marathe, Janhavi Katkar, Urmila M. Thatte

**Affiliations:** 1 Department of Pharmacology & Therapeutics, Seth GS Medical College & KEM Hospital, Parel, Mumbai, Maharashtra, India; 2 Institutional Ethics Committee, Seth GS Medical College & KEM Hospital, Parel, Mumbai, Maharashtra, India; 3 Department of Clinical Pharmacology, Seth GS Medical College & KEM Hospital, Parel, Mumbai, Maharashtra, India; Renal Division, Peking University First Hospital, CHINA

## Abstract

Protocol deviations (PDs) may jeopardize safety, rights, and welfare of subjects and data integrity. There is scarce literature and no guidelines for Institutional Ethics Committees (IECs) to process PD reports. The PD reports submitted to IECs from Jan 2011 to August 2014 were analyzed retrospectively. Types of studies reporting PDs, category and type of PDs, PD rate per participant, time of reporting PD since its occurrence and corrective actions stated by principal investigator (PI) for major deviations were noted. Out of 447 PDs from 73/1387 total studies received during study period, 402 were from 126 pharma studies. Investigator initiated studies and dissertations reported negligible PDs. Median number of PDs was 4 per protocol. Out of 447 PDs, 304 were related to study procedure, 87, 47 and 9 were from safety, informed consent document (ICD) and eligibility category respectively. The most common reason for PDs was incomplete ICD (22/47). Maximum study procedure related PDs were due to patient visiting outside window period (126/304). Thirty five of 87 PDs were due to missed safety assessment. The overall PD reporting rate per participant was 0.08. In 90% of reports, date of occurrence of PD was not specified. The median delay for reporting PDs after occurrence was 94 days. PDs classified as Major were 73% (323/447). The most common corrective actions stated by PI were participant counseling (85/323) and caution in future (70/323). The study findings emphasize the need for GCP training at regular interval of study team members. IEC have to be vigilant and visit sites frequently, take initiative and formulate guidelines regarding PD reporting.

## Introduction

Investigators have the responsibility of adhering to the protocol approved by the Institutional Ethics Committee (IEC). However, an investigator sometimes deviates from the study procedure approved by IEC. This is called a protocol deviation (PD), which is defined by the US FDA as an “unplanned excursion from the protocol that is not implemented or intended as a systematic change”[1]. Thus, any departure from the protocol without prior IEC approval is a protocol deviation. In this context the term protocol includes all the documents approved by the IEC. Protocol deviations may jeopardize in some way the safety, rights, and welfare of trial participants and data integrity of the study and therefore violate Good Clinical Practice (GCP) principles.

Various guidelines emphasize the importance of identifying protocol deviations and draw attention to the importance of prompt reporting of PDs. The International Conference on Harmonisation (ICH)—Good Clinical Practice (E6R1; Section 3.3.8a; 4.5.3) and Indian Good Clinical Practice (section 3.3.4: Communication with Ethics Committee) require that the investigator promptly reports to the ethics committee, the monitor and the sponsor deviations from or changes of, the protocol to eliminate immediate hazards to the subjects’[2,3]. The National institute of Health (NIH) further classifies PDs into minor and major PDs and protocol violations according to the impact of the deviation on patient safety and data integrity [4].

As this study was started in 2014, and the law governing conduct of clinical trials in the country was amended in 2005, we conducted an exhaustive literature search using Google search engine and PubMed for any original research articles /literature regarding protocol deviations from Jan 1^st^, 2004 to Dec 31^st^, 2014.We found only one systematic review that examined protocol violations reported from clinical trials of 80 clinical trials published in the Lancet, the New England Journal of Medicine, The Journal of the American Medical Association and the British Medical Journal [5]. Further, a site monitoring visit conducted by our IEC found some PDs had occurred at the study site which were not reported to the IEC [6]. Additionally, we could not find guidelines for IECs to process PD reports. With this background, this audit was conducted with the objectives of understanding the types of studies reporting PDs and the categories and types of PDs reported by a PI. We also wanted to assess the response of the IEC to these reports.

## Material and Methods

This was a retrospective observational study (EC/OA-94/2013) which was exempted from review (as per SOP 22 available at www.kem.edu/wp.content/uploads/2014/04/SOP-22pdf) by the Chairpersons of both the Institutional Ethics Committees of Seth GS Medical College and KEM Hospital, Mumbai, India who were independent of the institution and not authors of the audit. Both IECs are registered by the Drug Controller General of India as per rule 122DD 0f the Drugs and Cosmetic Rules (IEC-1: ECR/229/Inst./MH/2013 and IEC-2: ECR/417/Inst./MH/2013) and are also recognized by the Strategic Initiative for Developing Capacity in Ethical Review (SIDCER) since November 2012. We also obtained administrative approval from the Institutional Head.

PD documents were accessed from both IEC electronic records and the individual files of approved protocols. Information pertaining to the PD was extracted into an Excel spreadsheet. Patient records/ information were anonymized prior to extraction. We analysed protocol deviation reports from January 2011-August 2014 maintaining strict confidentiality of all the stakeholders. The period of study was based on availability of complete documentation for examination. Older study files were archived or destroyed depending on the IEC SOPs and newer files were relatively incomplete.

For the purpose of the study we classified PDs into four categories according to the domain that was affected by the deviation i.e. 1. Study procedure (e.g. participants receiving wrong treatment, participants seen outside window period, use of prohibited concomitant treatments, dosing outside protocol guidelines, missed visit/ assessment); 2. Safety (e.g. using prohibited concomitant treatments—prescribed dosing outside protocol guidelines, missed visit for safety assessment, failure to report Serious Adverse Event (SAE); 3.Informed Consent (failure to obtain informed consent, used Consent form not the current IEC-approved version, consent form does not include updates or information required by IRB, consent form missing), and 4.Eligibility (participants not meeting eligibility criteria, randomization of an ineligible participants, participants randomized prior to completing baseline Assessment, recruitment of participants prior to IRB approval of protocol). Deviations related to safety, informed consent and eligibility were considered as major deviations. Study procedure related deviations were further classified into major if they affected safety and well being of the participants, data integrity or in case the follow up visits were delayed by more than seven days. The deviations were considered minor if they did not fulfill the above criteria.

All seven authors took the decision regarding the categorization of PDs individually. We recorded our individual decision in the checklist provided. Whenever there was a disagreement, discussions were held between all authors till a consensus was reached.

The following were the outcome measures:

1. Types of studies reporting PDs
2. Category and types of PDs reported
3. PD rate per participant for each study type
4. Date of occurrence of PD
5. Corrective actions reported by PI
6. Actions taken by IEC.

The data was examined using descriptive statistics.

## Results

A total of 1387studies were under the oversight of the IECs during the study period, of which 818 (59%) were dissertations of postgraduate students, 406 (29%) were investigator-initiated studies, 126(9%) pharmaceutical company (pharma) sponsored clinical trials and 37(3%) studies were sponsored by government agencies. Of the total 1387 studies, 73 (5%) reported a total of 447 protocol deviations (median = 4, range 1–34). Of the total 73 studies, 63 (87%)were pharma-sponsored studies, 5 (7%)dissertations, 3 (4%)investigator initiated studies and 2(2%)government sponsored studies.

The overall PD reporting rate per participant was 0.08. The PD rate per participant for each study category is given in Table 1.

**Table 1:** PD rate per participant for each category.

	Total PDs	Total Participants	PD rate per participant (%)
**Pharma**	402	1103	0.36(36)
**Government**	3	3595	0.001(0.1)
**Investigator Initiated**	37	338	0.1 (10)
**Thesis**	5	369	0.01 (1)
**Total**	447	5405	0.08 (8)

Almost 90% PD reports did not mention the date of occurrence of the deviation in the reporting form and the median delay for reporting the deviation after occurrence (observed from those PDs reports that mentioned the date of occurrence) was 94 days (range 7–360).

The majority of PDs reported (402/447, 89.93%) were from pharma-sponsored studies followed by investigator-initiated studies (37/447, 8.28%). There was negligible reporting of PDs from theses of postgraduate students (5/447, 1.12%) and government sponsored studies (3/447, 0.67%).

PDs related to the study procedure (68%) formed the bulk of the types of PDs reported, followed by PDs related to safety (20%), ICD (11%) and eligibility (2%). The distribution of the categories of PDs reported is summarized in Table 2.

**Table 2:** Distribution of PDs according to different categories.

Type of studies / Category of PD [n(%)]	Study Procedure	Safety	ICD	Eligibility	Total
**Pharma**	266 (66.17%)	86(21.39%)	44 (10.94%)	6(1.49%)	402
**Government**	0	0	2 (66.67%)	1 (3.33%)	3
**Investigator Initiated**	36 (97.29%)	0	1 (2.70%)	0	37
**Thesis**	2 (40%)	1 (20%)		2 (40%)	5
**Total**	**304 (68%)**	**87 (19.47%)**	**47 (10.52%)**	**9 (2.01%)**	**447**

The distribution of various reasons for PD is summarized in Table 3. The most common PD in the study procedure related PDs was participant seen outside window period (41%).In almost 40% of safety related PDs, safety assessments were missed. Disturbingly in 47% of ICD related PDs, signature of key stakeholders were missing. There were multiple PDs of the same type reported from 16 pharma sponsored studies and 1 Govt. sponsored study. The most common repetitive PDs were “patient coming outside window period” and “missed assessment”.

**Table 3:** Summary of common reasons for occurrence of Protocol Deviations.

Category of deviation	Reasons of PD as reported by investigators	Number of deviations in each category
**Study Procedure N = 304**	Participant seen outside window period	126 (41.45%)
	Missed Assessment	92 (30.26%)
	Study procedure in protocol not followed	76(25.00%)
	Incomplete Case Record Forms	10 (3.29%)
**Safety N = 87**	Safety assessment not done at all	35(40.23%)
	Safety assessment not done as specified in protocol	31 (35.63%)
	SAE/ Adverse Event (AE) not reported	10 (11.49%)
	Prohibited medication taken	4 (4.60%)
	Stopped study medication	2 (2.30%)
	Wrong version of diary used	2 (2.70%)
**Informed Consent N = 47**	Failure to obtain informed consent properly	9 (19%)
	Wrong version used/ current version approved by IEC not used	16 (34%)
	Consent form does not have required signature	22 (47%)
**Eligibility N = 9**	Enrollment of ineligible participant	3 (33%)
	Randomization before baseline assessment	1 (11%)
	Study drug treatment of participant started before IEC approval	2 (22%)
	Recruitment of additional participants for the study	3 (33%)

Of the total 447 deviations reported 323 (73%) were major and 124 (27%) were minor. Of the 323 major deviations reported 70% were from pharma-sponsored studies. Of the total 447 deviations, all the safety (87), ICD (47) and eligibility (9) related PDs were considered as major as per our study definition (Total: 143). Of the 304 (447–143) remaining study procedure related PDs, 180 were considered major if they affected safety and well being of the participants, data integrity or in case the follow up visits were delayed by more than seven days. The detailed study type and study procedure category distribution of the major and minor deviations are given in Tables 4 and 5.

**Table 4:** Major and minor deviations for each study type.

Study Type (no. of deviations)	Major	Minor
**Pharma(402)**	281 (69.90%)	121 (30.10%)
**Government (3)**	3 (100%)	0
**Investigator Initiated (37)**	36 (97.30%)	1 (2.70%)
**Thesis(5)**	3 (60%)	2 (40%)
**Total (447)**	**323 (72.26%)**	**124 (27.74%)**

**Table 5:** Distribution of major and minor deviations for the study procedure category.

Reasons of PD as reported by investigators	Major	Minor
Participant seen outside window period (126)	58 (46%)	68 (54%)
Missed Assessment (92)	84 (91%)	8 (9%)
Study procedure in protocol not followed (76)	34 (45%)	42 (55%)
Incomplete Case Record Forms (10)	4 (40%)	6 (60%)
**Total (304)**	**180 (59%)**	**124 (41%)**

No corrective action was mentioned by the PIs in 43/323 (13%) major deviations. The most common corrective action described for major deviations was a statement that such deviations would not occur in future 70/323 (20%). The rest of the corrective actions taken by the PIs are summarised in Table 6.

**Table 6:** Distribution of corrective actions as reported by the PIs.

Corrective action/ category of deviation	Study procedure	Safety	ICD	Eligibility	Total (%)
Participant counseling	69	15	1	-	**85 (26)**
Study team training	4	26	11	2	**43(13.3)**
Investigators warned/ counseled	4	2	2	-	**8 (2.2)**
Reassurance that care will be taken in future	46	17	3	4	**70 (21.7)**
Revisit in case of missed assessment	15	8	-	-	**23 (7.1)**
Re-consent on ICD	-	-	16	-	**16 (5)**
Required signatures included	1	-	6	-	**7 (2.2)**
Patient dropout	16	6	5	1	**28 (8.7)**
No corrective action / Reported to sponsor	25	13	3	2	**43 (13)**
**Total**	**180 (55.73)**	**87 (26.93)**	**47 (14.55)**	**9 (2.79)**	**323**

All the PDs reported to the IEC were acknowledged. In 18 cases the IEC gave specific recommendations over and above the corrective actions given by the Principal investigator (PI). These included insisting on GCP training of study team, asking the status of participant terminated from the study and insisting on re-consenting of the study participants. In one of the studies, where more participant over and above that approved were recruited, the IEC directed that the data of these additional participants should not be used for analysis.

## Discussion

Our audit revealed that only 5% of the total studies under IEC oversight during the study period (January 2011 to August 2014) reported PDs to our IEC with a median of 4 PDs per protocol, The maximum reports were from pharma-sponsored studies and very few from dissertations/ Government sponsored studies. More than half the PDs were major deviations and there were considerable delays in reporting PDs.

It is difficult to comment whether the overall PD rate per participant of 8% as seen in our study is high or low. Unfortunately, there is no defined “expected” PD rate. Other studies have described a PD rate ranging from 16% to 25% per protocol reporting PDs [5]. There is one study that mentions that PDs in more than 10% of enrolled patients is “unacceptable” [5]. Ours was below this. Similarly there are no standards laid down for “permitted” delay in reporting a PD after it occurs. Our study found a median delay of 94 days in reporting PDs to the IEC.

The ICH-GCP guidelines [2], under the section 4.5 “Compliance with protocol” clearly state that ‘the investigator should document and explain any deviation from the approved protocol’. Although there were 1387 research studies under the oversight of the IEC during the study period, only 73 (5%) actually reported PDs. It is impossible to ascertain whether PDs did not occur or they were just not reported. Although pharma-sponsored studies formed only 9% of the studies that were under oversight of the IEC during the study period and only 50% of these studies reported PDs, they constituted 90% of the total deviations reported. This high rate of reporting may be due to the monitoring that occurs for sponsored studies while this does not happen in either investigator-initiated studies or postgraduate thesis. The median delay in reporting PDs in Pharma sponsored studies was higher than in any of the other categories and these reports were bunched together, suggesting that the PD reporting occurred only after detection at monitoring by the sponsor.

The IEC plays a major role in reviewing and monitoring academic research along with pharmaceutical studies, and the same ethical standards have to be applied to all research in an institute. Studies submitted by postgraduate students of the institution are often time bound and lack internal monitoring systems. Under- reporting of PDs could be due to ignorance, lack of or inadequate GCP training of investigators [7] (especially the PG students) that may result in failure to recognize PDs and report them in time. Of the 5 PDs reported from the dissertation projects, 3 were recruitment of additional participants without IEC approval. Of these 5 deviations, 4 were identified by the IEC while going through the completion report. The IEC then issued a directive asking the PI to report the PD. From this it appeared that although deviations may be occurring in dissertation projects they may not be reported. Thus, there is a definite need for institutions to take steps towards sensitizing the postgraduates for the awareness of ethical principles as well as guidelines that have to be followed while conducting any type of research. We found similarly poor PD reporting from Government funded studies, which illustrates that government support is often only financial with no “sponsor” role played by the funding agency. The investigator then has to play the role of a sponsor and must put in place adequate monitoring systems to ensure GCP compliance.

Any clinical study is a partnership between the study team members and participants. The most common reason stated for study procedure deviations was participant being outstation or at his/her native place and hence not being able to report on the date given by investigators for follow up. Most times patients prefer getting enrolled in a study if the site is close to their residence [8]. Thus, patients from out of town who still enroll for studies may end up in being defaulters on the protocol which may on occasion have a serious impact on either safety and well being of the participant or data integrity. The PI needs to ensure that participants have understood the protocol and are ready to comply with it, should be enrolled for the study. Once enrolled, the study team should ensure compliance to the study protocol by regular reminders. Some of the solutions suggested by *Lamberti*, *et al (2012)* to improve adherence to the protocol by participants are improving rapport and contact with the patients, reinforcing value of participation, reminders *via* telephone / email etc [9].

“Missing data points” caused by the missed follow up visits can affect safety of the participants but it is also a challenge during analysis and interpretation of the results. Missing data leads to biased results, flawed interpretation and loss of statistical power [10].Hence, it is recommended by Harel*et al* [11]“that with any applied research and in particular RCTs, the best thing to do with regard to missing data is to avoid it. The second best thing is to plan for it, understand it and address it with appropriate modeling techniques beforehand in the protocol”.

Safety assessment is essential to understand the intervention as much as to protect the research participant. In our study we found 87 PDs related to safety assessment, of which 35 times the study team had failed to either perform important safety investigations like fundoscopy, liver Function test (LFT), Magnetic Resonance Imaging (MRI), etc. or had done them at the wrong times (n = 31). This too can have serious impact on both data as well as safety of the research participant. Again the importance of training of the whole team is highlighted by these observations [12,13]. Investigators also need training on time lines for safety reporting as these have undergone several changes, in India to avoid deviations related to delayed reporting of AE/SAE [14].

The informed consent procedure forms the backbone for any type of clinical research involving human participants. A previous study has reported that a large proportion of warning letters (almost 50% in one of the studies) issued by the US FDA to investigators were for informed consent violations over the period 2005 to 2012 [15,16]. The common violations for which warning letters were issued included failure to obtain consent before screening, backdating consent forms by the clinical investigator and using a consent form of a different study/ different version. We noted that out of 47 ICD related violations, re-consent was obtained only on 16 occasions (34%). The Ethics Committee is expected to ensure adequate oversight so that informed consent is appropriately taken.

One of the important challenges for investigators is to recruit the required number of participants in a given time period. While achieving this goal study team members can become restless and manipulative [17] which might jeopardize the safety of ineligible participants. Nine PDs reported to our IEC relating to recruitment of ineligible participants could have major implications on participant safety and data integrity. The PI needs to give attention to this issue by strictly following eligibility criteria as stated in protocol and providing adequate training to the inexperienced study coordinators to avoid these violations.

PIs have described patient education/ counseling and study team training as corrective actions for most PDs. Although there was no reporting of harm for major deviations by the investigators these could have affected data integrity of the study. However this is difficult to prove. Re-occurrence of a PD after a statement of corrective action was seen in nearly all studies. It was difficult to ascertain if these corrective actions were really implemented by the investigators. The IEC needs to ensure that these corrective actions are taken either by documented evidence or site monitoring visits.

Although ICH-GCP was introduced to set standards for clinical trials on new drugs, it is evident that if the Declaration of Helsinki has to be adhered to, then the principles of GCP need to be applied to all types of clinical research. Hence our study which found high PD reporting rate from pharma sponsored studies but very poor reporting rate from academic studies has important pertaining both to the investigators as well as IECs. Investigators need to work vigilantly to avoid protocol deviations altogether or report and take appropriate corrective steps in case they occur. Training study team members periodically, establishing good rapport through communication with participants, having site specific SOPs for protocol deviations reporting and internal audit by investigator himself of his site will go a long way in minimizing occurrence of PDs which are avoidable. Postgraduate students and investigators should be sensitized for reporting of protocol deviations in these studies.

IECs too have to be more proactive and should lay down guidelines for deviations reporting timelines, ask for periodic log of deviations and conduct site monitoring visits. Protocol deviations should be reviewed by IEC as per written standard operating procedures. Our study has a few limitations including being a retrospective audit and lacking information on actual impact of the PDs on the patient safety and the final study report.
